# Hybrid strategy in compact tailoring of multiple degrees-of-freedom toward high-dimensional photonics

**DOI:** 10.1038/s41377-025-01857-3

**Published:** 2025-04-21

**Authors:** Shiyun Zhou, Lang Li, Liliang Gao, Zhiyuan Zhou, Jinyu Yang, Shurui Zhang, Tonglu Wang, Chunqing Gao, Shiyao Fu

**Affiliations:** 1 School of Optics and Photonics, Beijing Institute of Technology, 100081 Beijing, China; 2 Key Laboratory of Photoelectronic Imaging Technology and System, Ministry of Education of the People’s Republic of China, 100081 Beijing, China; 3 Key Laboratory of Information Photonics Technology, Ministry of Industry and Information Technology of the People’s Republic of China, 100081 Beijing, China; 4 Key Laboratory of Quantum Information, University of Science and Technology of China, 230026 Hefei, China; 5 Synergetic Innovation Center of Quantum Information & Quantum Physics, University of Science and Technology of China, 230026 Hefei, China; 6 Beijing National Research Center for Information Science and Technology, School of Integrated Circuits, Tsinghua University, 100084 Beijing, China; 7 National Key Laboratory on Near-surface Detection, 100072 Beijing, China

**Keywords:** Optical physics, Optical techniques

## Abstract

Tailoring multiple degrees-of-freedom (DoFs) to achieve high-dimensional laser field is crucial for advancing optical technologies. While recent advancements have demonstrated the ability to manipulate a limited number of DoFs, most existing methods rely on bulky optical components or intricate systems that employ time-consuming iterative methods and, most critically, the on-demand tailoring of multiple DoFs simultaneously through a compact, single element—remains underexplored. In this study, we propose an intelligent hybrid strategy that enables the simultaneous and customizable manipulation of six DoFs: wave vector, initial phase, spatial mode, amplitude, orbital angular momentum (OAM) and spin angular momentum (SAM). Our approach advances in phase-only property, which facilitates tailoring strategy experimentally demonstrated on a compact metasurface. A fabricated sample is tailored to realize arbitrary manipulation across six DoFs, constructing a 288-dimensional space. Notably, since the OAM eigenstates constitute an infinite dimensional Hilbert space, this proposal can be further extended to even higher dimensions. Proof-of-principle experiments confirm the effectiveness in manipulation capability and dimensionality. We envision that this powerful tailoring ability offers immense potential for multifunctional photonic devices across both classical and quantum scenarios and such compactness extending the dimensional capabilities for integration on-chip requirements.

## Introduction

Recent decades have witnessed the rapid development of laser field manipulation, where on-demand tailoring of multiple degrees-of-freedom (DoFs) emerging as a leading area of research^1–7^. The ability to tailor a greater number of DoFs determines the creation of complex optical fields with high dimensionality, which is critical for various applications, including next-generation laser^8^, optical tweezers^9^, quantum computing^10–13^, and holography^14^. Despite that previous techniques have made substantial progress in tailoring limited DoFs, such as polarization^15,16^, amplitude^17^, and initial phase^18^, they still face inherent limitations in dimensionality that hinder the achievement of high-dimensional and multiple DoFs manipulation simultaneously. Orbital angular momentum (OAM), a new DoF, has been considered to enhance tailoring dimensionality due to its unbounded orthogonality^19–22^. However, the phase-only modulation for multiple OAM modes lead to unpredictable overlaps^23^, which complicates the integration in single elements and poses challenges to cope with other DoFs. As additional DoFs are introduced, the cost and complexity rapidly grow, leading to higher computational demands and reduced tailoring precision. To overcome these challenges, current schemes for manipulating multiple DoFs often rely on bulky optical components or intricate systems that employ time-consuming iterative methods, sacrificing system simplicity or speed to compensate for the increased complexity. Driven by a growing demand for high-dimensional on-chip integration^24,25^, there is a highly desirable for on-demand tailoring of multiple DoFs through compact and lightweight devices^26^ that are simple, fast and accurate. Addressing this demand is essential for advancing capabilities of photonic technologies and unlocking new possibilities for innovative applications^27^.

In this paper, we propose an intelligent hybrid strategy that enables the compact tailoring of multiple DoFs as wave vector, initial phase, spatial mode, amplitude, OAM, and SAM, simultaneously, thus constructing a high-dimensional optical fields. Our proposal overcomes the limitation of time-consuming iterative methods and advances in phase-only property, facilitating integration onto compact device. In details, a function inspired by scalar diffraction theory is established to determine the wave vector and initial phase effectively. Next, an intelligent deep neural network is introduced to derive a phase-only hologram from complex amplitude information, completing the on-demand manipulation of OAM and amplitude in different spatial mode. Finally, we incorporate the geometric phase, achieving an overall vectorial manipulation of SAM. The advancing phase-only property of our proposal can be easily integrated into various diffractive optical elements (DOEs). Here we experimentally demonstrated by fabricating a compact metasurface sample for six-DoFs, 288-dimensional manipulation as a proof-of-principle. Experimental results align closely with simulations, yielding average mean square errors (MSE) of 0.0036 for total angular momentum (TAM) spectrum measurement under a quantitative evaluation. This research showcases a compact, customizable, and intelligent hybrid strategy for tailoring multiple DoFs in high-dimensional laser fields. We firmly believe that this work significantly enhances the capabilities of multiple DoFs tailoring, extends the dimensional capabilities of photonics and paves the way for on-chip scenarios.

## Results

### Concept overview

**Fig. 1:**
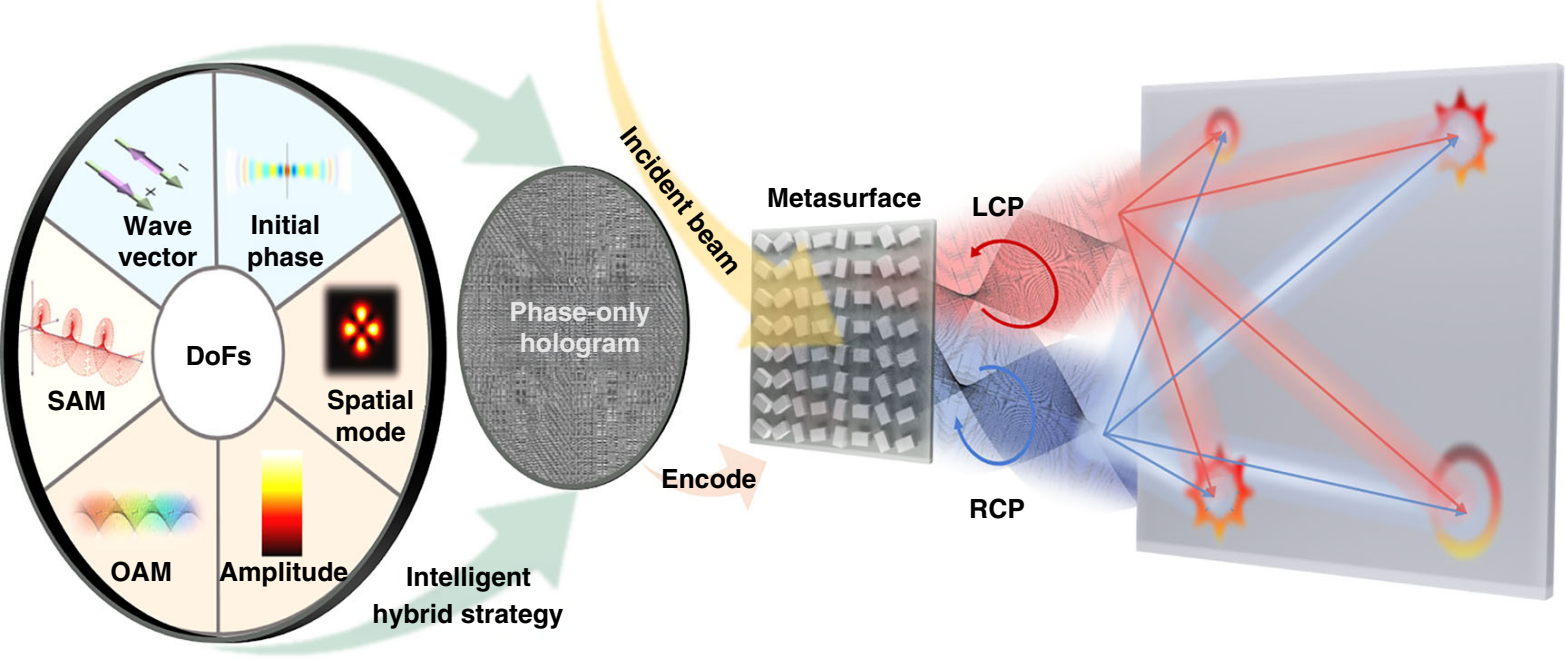
Schematic diagram of the proposal. The functions of arbitrary manipulations over wave vector, initial phase, spatial mode, amplitude, OAM, and SAM are integrated into a phase-only hologram, which is then encoded onto a metasurface, transforming the incident beam to a customized high-dimensional structure field

The schematic diagram of our proposal is shown in Fig. 1. Our intelligent hybrid strategy enables the simultaneous on-demand tailoring of six DoFs as wave vector, initial phase, spatial mode, amplitude, OAM and SAM. The resulting phase-only hologram is then encoded onto a metasurface to accomplish the manipulation. This single element allows for a compact tailoring of the incident beam, which can introduce various phase modulation for left and right circular polarizations (LCP and RCP), and then simultaneously incorporate the two circular polarization of corresponding diffraction orders. These outputs exhibit single or superposed OAM modes, each characterized by distinct amplitude and initial phase, thus forming varied spatial modes.

**Fig. 2:**
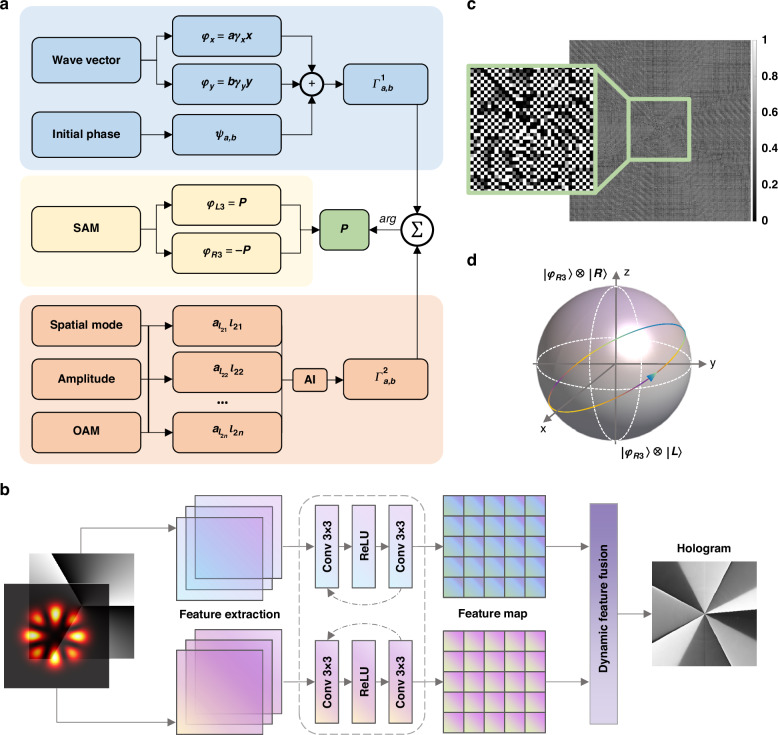
Principles of the intelligent hybrid strategy for multiple DoFs manipulation. **a** The work flow of intelligent hybrid strategy for generating the modulated DOE phase profiles. The desired phase-only profile $P$ consists of three components: ${\varGamma }_{a,b}^{1}$, which pertains to the manipulation of wave vector and initial phase; ${\varGamma }_{a,b}^{2}$, derived from an intelligent deep neural network designed for tailoring superimposed OAM mode and corresponding amplitude; and the overall geometric phase ${\varphi }_{L3}$ and ${\varphi }_{R3}$ for SAM tailoring. **b** The architecture of the deep neural network for superposed OAM and amplitude tailoring. By training on the complex amplitude information through the strategies of feature extraction and a dynamic feature fusion, a mapping between the target superposed OAM beam and phase-only transmittance function can be established. The trained model allows direct hologram output of target OAM mode corresponding with amplitude distribution, forming different spatial mode. **c** A phase distribution instance of the finally obtained *Γ*, which is then encoded onto a metasurface to accomplish six-DoFs, 288-dimensional beam tailoring, where the center zooms for better view. **d** Principle of overall geometric phase modulation. By introducing left and right circular components ${\varphi }_{L3}$ and ${\varphi }_{R3}$ toward the polarization angle ${|L}{{\rangle}}$ and ${|R}{{\rangle}}$ of the generated beam, one can achieve the modulation of SAM

As illustrated in Fig. 2a, the principle of our intelligent hybrid strategy comprises three main stages, with each stage contributing to the overall manipulation of six DoFs in high-dimensional optical fields. The strategy integrates wave vector and initial phase tailoring, spatial mode generation, multiple OAM with various amplitude customization, and the incorporation of geometric phases for SAM manipulation. The synergy of these elements allows for efficient and precise high-dimensional field tailoring. The workflow begins with tailoring the wave vector. For each diffraction order, the initial phase, spatial mode, OAM, and amplitude are customized. The initial phase can be directly tailored by superimposing the transmittance function. For coping with OAM and the corresponding amplitude, common mode-iterative methods suffer from time-consuming limitations thus hindering the further high-dimensional construction^28^. Inspired by the powerful abilities of artificial intelligence in reverse design, we propose a deep neural network, enabling the efficient creation of phase-only holograms for OAM and amplitude manipulation. Once the manipulation mechanism for a single diffraction order is established, we then extend this manipulation to multiple diffraction orders to form a coherent, multi-level optical field. Finally, we incorporate the geometric phase, which allows for a smooth integration of SAM, combining it with the wave vector, initial phase, spatial mode, amplitude, and OAM, thus facilitating a fully tailored, vectorial optical field.

Considering a beam located in position (*a*, *b*) in a Cartesian coordinate (*x*, *y*), with *a* and *b* diffraction orders in $x,{y}$ direction, respectively, one can form a phase-only transmittance function ${\varGamma }_{a,b}^{1}$:

1 $${\varGamma}_{a,b}^{1}=\exp \left[{{i}}\left(a{\gamma}_{x}x+b{\gamma}_{y}y\right)\right]\exp \left({{i}}{\psi }_{a,b}\right)$$

where ${\gamma }_{x}$ and$\,{\gamma }_{y}$ correspond to spatial frequency along $x,{y}$ axes. ${\psi }_{a,b}$ is the initial phase at diffraction order (*a*, *b*). Eq. (1) enables us to tailor the wave vector by setting the parameters $a,{b}$, tailor initial phase by setting parameter ${\psi }_{a,b}$. Then, we utilize an intelligent deep neural network to derive a phase-only transmittance function that manipulates superposed OAM mode with selective intensity distributions, manipulating both OAM and amplitude on-demand, resulting in various spatial mode. Previous studies have proved that multiple superposed OAM beam cannot be directly generated through a phase-only hologram^29^. Existing iterative methods are limited by slow computation speed, lack of on-demand customization, and incomplete convergence. Inspired by the robust capabilities of deep learning^30–32^, we develop a convolutional neural network specifically designed to address the challenges of generating superposed OAM holograms. This network employs a multi-layer perceptron to extract high-level representations, compensating for intensity losses caused by overlapping modes. Additionally, a dynamic feature fusion strategy integrates these extracted characteristics, allowing for the generation of phase-only holograms generation and precise manipulation of multiplexed OAM states. The architecture is depicted in Fig. 2b. The target complex amplitude $X\left({a}_{l},l\right)$, determined by orthogonal OAM modes with varying amplitudes:

2 $$X\left({a}_{l},l\right)=\mathop{\sum }\limits_{l=-\infty }^{+\infty }{a}_{l}\exp ({{{i}}l}\varphi )$$

where ${a}_{l}$ represents the amplitude distribution and *l* denotes the OAM orders. This complex amplitude encapsulates both intensity and phase information of the optical field. It serves as the input to the network, which undergoes down-sampling through a multi-layer perceptron consisting of two $3\times 3$ convolutional layers connected by an activation function, extracting relevant features. The transformation of the input into a high-level representation is captured as a feature map:

3 $${Y}_{x,y}\left({a}_{l},l\right)={{\rm {ReLU}}}\left(\mathop{\sum}\limits_{m=0}^{M-1}\mathop{\sum }\limits_{n=0}^{N-1}{X\left({a}_{l},l\right)}_{x+m,y+n}{K}_{{mn}}+c\right)$$

In Eq. (3), ${Y}_{x,y}$(${a}_{l},l$) denotes the feature value at position ($x,\,y$), and $K$ is the convolutional kernel of size $M\times N$ (set to 3 in this case). The indices $m$ and $n$ refer to the kernel positions. ReLU is the activation function to introduce non-linearity, with $c$ a constant parameter. Following feature extraction, we employ a dynamic feature fusion strategy to combine the extracted characteristics, which can be described as

4 $${D}_{{x}^{{\prime} },{y}^{{\prime} }}\left({a}_{l},l\right)=\left(1+p\right)\left(1-q\right){Y}_{x,y}+p\left(1-q\right){Y}_{x+1,y}+\left(1-p\right)b{Y}_{x,y+1}+{pq}{Y}_{x+1,y+1}$$

Here, ${D}_{{x}^{\prime} ,{y}^{\prime}}$ denotes the feature value at position (${x}^{\prime} ,{y}^{\prime}$) under dynamic sampling scales, ${Y}_{x,y}$ is the nearest pixel of ${D}_{x,y}$ in the original scale feature map, $p,\,q$ represent the proportional horizontal and vertical distances of $x,\,y$ relative to the closest pixel ($x,\,y$). This formulation effectively performs bilinear interpolation by weighting the four nearest neighbor features based on their proportional distances from the dynamic sampling point. Such an approach enables the network to adaptively fuse multi-scale features, ensuring both fine-grained details and higher-level contextual information are preserved. Finally, we achieve the output hologram ${\phi }_{{{\rm {holo}}}}$ through a weighted summation, which is represented as

5 $${\phi }_{{{\rm {holo}}}}=\sum _{n}{{{\rm {Conv}}}}_{1\times 1}\left({D}_{n}\left({a}_{l},l\right)\times {w}_{n}\right)$$

where ${{{\rm {Conv}}}}_{1\times 1}$ is a convolutional layer with a kernel size of $1\times 1$. ${D}_{n}$ represents the output feature fusion, and ${w}_{n}$ is the weight coefficients (initially set to 1 and optimized during training) (see Supplementary Note 1 for more details). This dynamic feature fusion, combined with the final 1 × 1 convolution, allows the network to effectively merge information from multiple scales and channels, enhancing the quality of the output hologram and ultimately improving the overall performance of the system. Thus, the complex transmittance function is given by

6 $${\varGamma}_{a,b}^{2}=\exp \left({{i}}{\phi }_{{{\rm {holo}}}}\right)$$

This formulation enables the high-dimensional synergistic manipulation over superposed OAM mode with different amplitude, forming various spatial mode under different incident beam.

Next, building on the foundations established above, we integrate the phase-only transmittance functions to construct a hierarchical architecture underlying a hybrid strategy. As detailed in Fig. 2c, this approach enables the realization of a customizable multiple DoFs, and the phase distribution $P$ reads:

7 $$P={{\arg }}\left[\sum _{\left(a,b\right)}{A}_{a,b}\cdot \left({\varGamma }_{a,b}^{1}\cdot {\varGamma }_{a,b}^{2}\right)\right]$$

where ${\arg }(\cdot )$ denotes taking the a rgument. For optimizing the transmittance function $P$, the optimal coefficient ${A}_{a,b}$ and an iterative method are introduced. The process begins with an initial set of coefficients ${\{A}_{a,b}^{\left(0\right)}\}$. During each iteration $m$, we update the coefficients ${A}_{a,b}^{(m+1)}$ by minimizing the difference between the diffraction field $E{\prime}$, which generated by a Gaussian beam through the current modulation phase ${P}_{a,b}^{(m)}$, and the target field $E$. This optimization is performed using gradient descent, with the update rule defined as

8 $${A}_{a,b}^{\left(m+1\right)}={A}_{a,b}^{\left(m\right)}-\eta \frac{\partial L\left({E}^{{\prime} },E\right)}{\partial {A}_{a,b}^{\left(m\right)}}$$

Here, $\eta$ represents the learning rate, and $L$ is the loss function, calculated as the mean squared error between $E{\prime}$ and $E$. The iteration process continues until the difference between $E{\prime}$ and $E$ falls below a predefined threshold or the maximum number of iterations is reached.

Finally, to tailor SAM, we incorporate the geometric phase^33–36^. By setting the orientation of the fast axis of the birefringent medium at each position ($x,\,y$), one can introduce different phase modulation for the LCP and RCP components of the incident beam, respectively. The phase profile $P(x,y)$ is responsible for changing the polarization angle, as shown in Fig. 2d, resulting in the modulated output field:

9 $$\left|E\right.{{\rangle }}={\exp \left[i{\varphi }_{R3}\left(x,y\right)\right]E}_{L}\left|R\right.{{\rangle }}+\exp [i{\varphi }_{L3}(x,y)]{E}_{R}\left|L\right.{{\rangle }}$$

with $$\left|R\right.\rangle ={[\begin{array}{cc}1 & i\end{array}]}^{{{T}}},\,\left|L\right.\rangle ={[\begin{array}{cc}1 & -i\end{array}]}^{{{T}}}$$ corresponding to left and right circular polarizations. This effectively exchanges the LCP and RCP components of the incident light field, introducing additional geometric phases of ${\varphi }_{R3}=-P$ and ${\varphi }_{L3}=P$, thereby achieving anisotropic polarization modulation and thus tailoring SAM. Notably, a key advantage of our approach lies in its phase-only property, which enables high compatibility with various diffractive optical devices. It can be implemented using birefringent materials, including but not limited to polymeric liquid crystals, metasurfaces, and other anisotropic modulation dielectrics.

### Metasurface design and experiment demonstration

In our proof-of-principle demonstration, the tailored six DoFs include wave vector, initial phase, OAM, amplitude, spatial mode, and SAM. Specifically, we target the wave vector across four diffractive orders, located at positions (1, 1), (−1, 1), (1, −1), and (−1, −1). For the (1, 1) order, it is an LCP beam with a superposed OAM modes [3, −1, −5] is assigned. The corresponding amplitude distribution of these OAM modes is set as [0.3, 0.06, 0.64]. For the (−1, 1) order, the OAM mode for LCP component is set to 2 with a geometric phase of 0, while the RCP component is set to -1 with a geometric phase of $-\pi /4$. For the (1, −1) order, the OAM mode for LCP component is set to 1 with a geometric phase of $\pi /4$, and the RCP component is set to −2 with a geometric phase of 0. Finally, for the (−1, −1) order, an RCP beam is assigned a superposed OAM with orders [−3, 1, 5] and the amplitude distribution of [0.3, 0.06, 0.64]. As summarized in Table 1, the overall tailoring dimensions include four for wave vectors, and two for the spatial mode. For each wave vector, the maximum tailoring dimensions are two for initial phase, three for OAM, three for amplitude, and two for polarization, resulting in a total of 288 dimensions.

**Table 1:** Tailoring DoFs and the calculation of dimensions

DoFs	Dimensions (Dim.)						Max. dim.
Wave vector	(1, 1)	(−1, 1)		(1, −1)		(−1, −1)	4
Spatial mode	Linear polarization incident						2
	Circular polarization incident (L)						
		Circular polarization incident (R)					
	Superposed	Single		Single		Superposed	
Initial phase	–	0	$-\frac{\pi }{4}$	$\frac{\pi }{4}$	0	–	2
OAM	[3, −1, −5]	2	−1	1	−2	[−3, 1, 5]	3
Amplitude	[0.3, 0.06, 0.64]	1	1	1	1	[0.3, 0.06, 0.64]	3
SAM	LCP	L	R	L	R	RCP	2
Total: 4 × 2 × 2 × 3 × 3 × 2 = 288							

Toward future integrated on-chip scenarios, here the metasurface is employed as the carrier of the proposed intelligent hybrid strategy, achieving six-DoFs, 288-dimensional manipulation. The Jones matrix expression for the designed metasurface can be derived as follows:

10 $$J\left(x,y\right)=\left[\begin{array}{cc}\cos P\left({x},{y}\right) & -{{i}}\sin P\left({x},{y}\right)\\ -{{i}}\sin P\left({x},{y}\right) & -\cos P\left({x},{y}\right)\end{array}\right]$$

By accurately encoding the Jones matrix onto the metasurface, we enable efficient, simultaneous tailoring of the wave vector, initial phase, spatial mode, amplitude, OAM, and SAM, constructing the complex high-dimensional optical fields with a single, versatile, compact device.

**Fig. 3:**
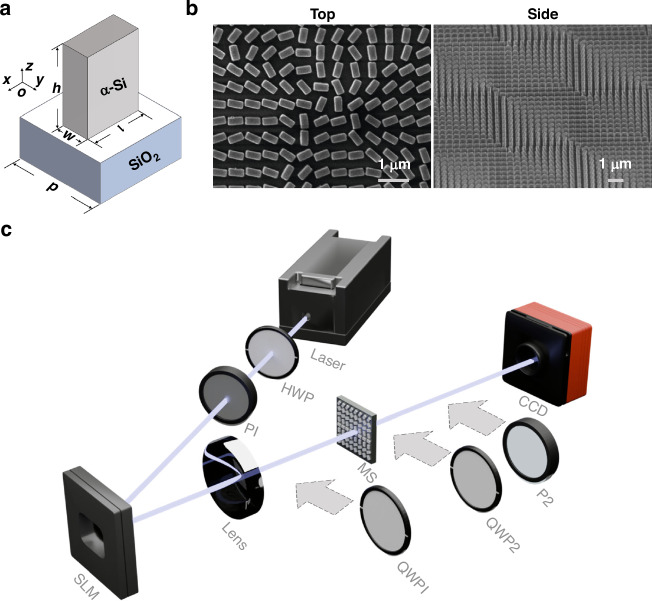
The fabricated metasurface and experimental setup. **a** Schematic illustration of an amorphous silicon nanofin on a SiO_2_ substrate. **b** The scanning electron microscopy images of the fabricated metasurface sample in top and side views. **c** The experimental setup. P1 and P2 linear polarizers, HWP half-wave plate, SLM spatial light modulator, Lens plano-convex lenses with focal length 300 mm; MS metasurface, QWP1 and QWP2 quarter-wave plates, CCD infrared CCD camera. Notably, QWP1, QWP2, and P2 are introduced for further tailoring performance evaluation

As shown in Fig. 3a, our fabricated metasurface is composed of arrays of amorphous silicon (α-Si) nanopillars located on a SiO_2_ substrate. By adjusting the length, width, and orientation angle of each nanopillar, independent phase distributions of the two orthogonal circularly polarized components can be encoded to the metasurface. Each nanopillar satisfies the half-wave condition, allowing geometric phase modulation by arranging the orientation angle. The dimensions and orientation angle of the nanopillar at each pixel are determined by analyzing the eigenvalue and eigenvector of the corresponding Jones matrix. The parameters are optimized using finite-difference time-domain (FDTD) methods. The finally designed six-DoFs 288-dimensional sample is composed of 1080 × 1080 nanopillars (size: 0.7 mm^2^). At the working wavelength of 1617 nm, the highest performance efficiency is achieved with each nanopillar having a period of 650 nm, a height of 1020, 513.3 nm in length, and 214.3 nm in width (see Supplementary Note 2 for more details). Under the above design, the metasurface is fabricated with the use of the e-beam lift-off method, and the scanning electron microscopy images of our fabricated metasurface in top and side views are illustrated in Fig. 3b.

The experimental setup is depicted in Fig. 3c. A half-wave plate (HWP) together with a polarizer (P1) is employed to generate incident horizontally linear polarization. Then, the beam is reflected by the spatial light modulator (SLM) and subsequently focused onto the fabricated metasurface through a convex lens with a focal length of 300 mm. Note that, the SLM is utilized to encode various anti-spiral-phases for further OAM analysis of the tailored high-dimensional field. During the tailoring, the SLM simply acted as a reflector. An infrared CCD camera is used to capture the intensity distribution. Additional optical elements, including two quarter-wave plates (QWP1, QWP2) and a polarizer (P2), are introduced when evaluating tailoring performance.

**Fig. 4:**
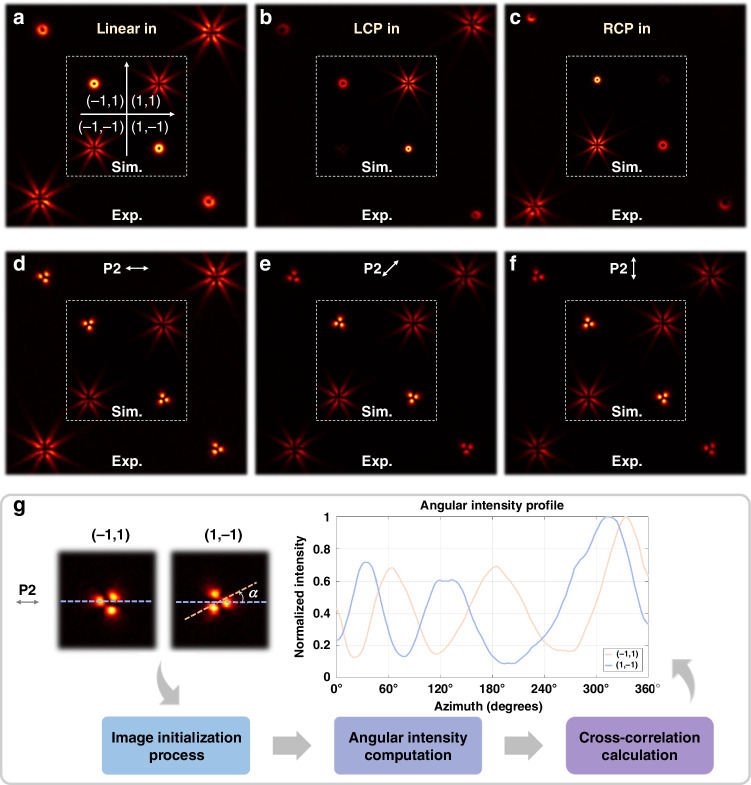
Experimental and simulated results of six-DoFs, 288-dimensional tailoring. **a**–**c** Intensity profiles of the tailored field when linear and circular polarizations are incident, respectively. The incident left and right circular polarizations are generated by using an additional −45°/45° arranged quarter-wave plate (QWP1). **d**–**f** Experimental and simulated results of (a) after passing through an additional 0°, 45°, and 90° arranged linear polarizers (P2), respectively. **g** The initial phase analysis. By calculating the rotational deviations result from the tailored initial phase at the (−1, 1) and (1, −1) diffraction orders, the accuracy of the phase tailoring can be validated by measuring the rotation angle $\alpha$

### Tailoring performance

The tailoring results are visualized in Fig. 4. Under a quantitive evaluation, the specific modulation performances of the experimental results in six-DoFs, 288-dimensional tailoring are outlined as follows:

i. *Wavevector*: The variations of diffraction orders located at (1, 1), (−1, 1), (1, −1), and (−1, −1) demonstrate an effective tailoring of the wavevector, as shown in Fig. 4a.
ii. *Spatial mode*: Fig. 4a–c displays the intensity profiles of the tailored optical field under different incident polarizations, including linear and circular polarization. Each profile contains single and superposed two kinds of patterns. Note that, the left and right circular polarization incident is accomplished by placing a QWP1 before the metasurface.
iii. *SAM*: Fig. 4d–f presents the polarization distributions, which are obtained by introducing another polarizer P2 in front of the CCD, oriented sequentially at 0°, 45°, and 90° arranged. The visualization indicates the two-dimensional nature of SAM.
iv. *Initial phase*: A challenge is how to verify the phase tailor performance. The target-tailored initial phases at diffraction orders (−1, 1) and (1, −1) can be represented as follows:
  11 $${|\varPsi {{\rangle }}}_{(-1,1)}=\frac{\sqrt{2}}{2}\left({|L}{{\rangle }}\left|2\right.{{\rangle }}+\,\left|R\right.{{\rangle }}|-1{{\rangle }}\cdot \exp \left({{i}}\left(-\frac{\pi }{4}\right)\right)\right)$$
  12 $${\left|\varPsi \right.{{\rangle }}}_{(1,-1)}=\frac{\sqrt{2}}{2}\left({|L}\rangle |1{{\rangle }}\cdot \exp \left({{i}}\frac{\pi }{4}\right)+{|R}{{\rangle }}|-2{{\rangle }}\right)$$
  Since the initial phase difference is reflected on the rotation angle $\alpha$ at the polarization origin in positions (−1,1) and (1, −1)^37^. By measuring the discrepancy, the carried initial phases can be calculated as:$2\cdot \alpha +(\alpha +\pi /4)=-\pi /4$, the rotation angle is derived as $\alpha =-30^\circ$, thus the pattern distribution at the (−1, 1) and (1, −1) positions should rotate by 30°. For quantitative evaluation, the flowchart for experimentally rotation angle calculation is given in Fig. 4g. Measurements under passing through 0°, 45°, and 90° arranged linear polarizers (P2) yielded an average rotation angle of 30.6°, demonstrating successful modulation of the carried initial phase to *π*/4 (see Supplementary Note 3 for more details).

**Fig. 5:**
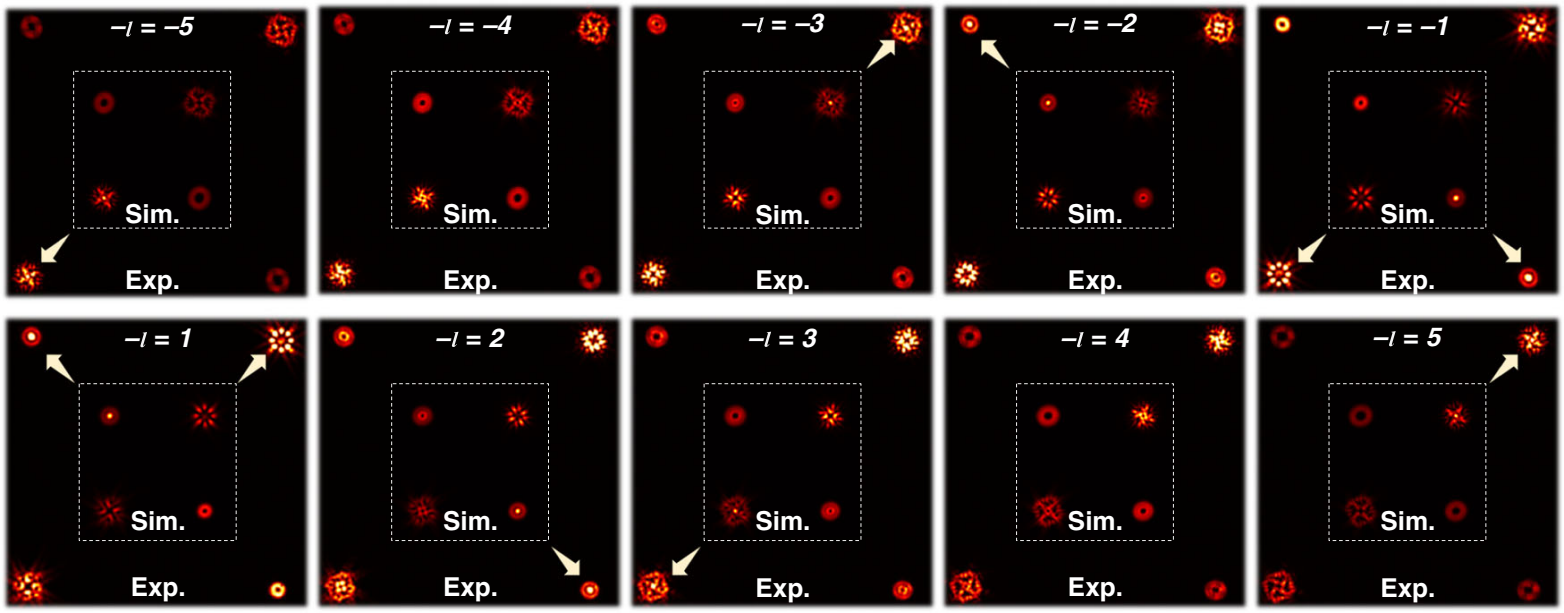
Experimentally captured back-converted patterns for OAM mode analysis. The orders of the back-converting spiral phases are labeled at the top of each inset

**Fig. 6:**
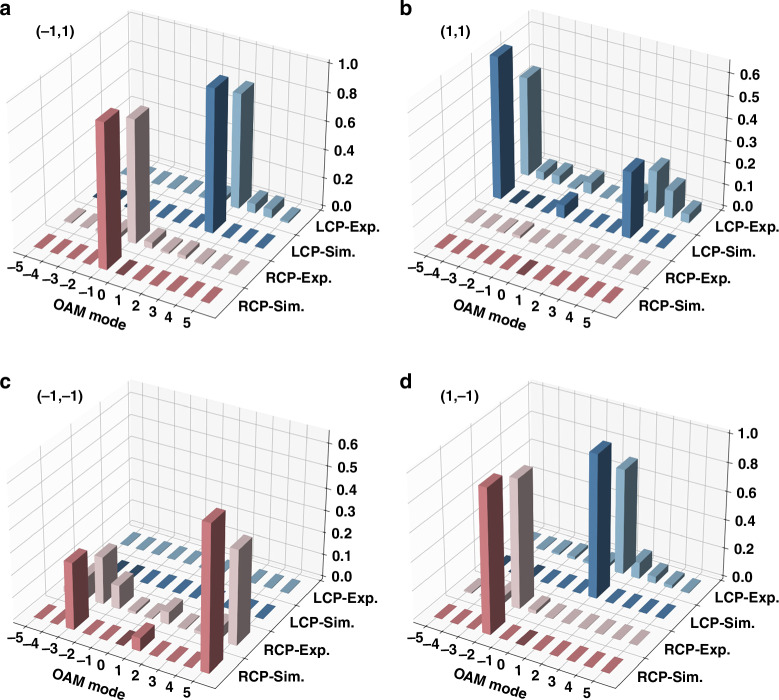
Quantitative amplitude analysis. **a**–**d** are corresponding to four diffraction orders, showing OAM modes ranging from −5 to 5, with SAM represented by LCP and RCP components in blue and red bars, respectively
v. *OAM*: By encoding various spiral phases on SLM, following OAM back conversion theory^38,39^, the OAM modes can be analyzed. The principle of back conversion can be explained as follows: if a -*l*th order spiral phase is encoded, the OAM state $|l{{\rangle }}$ in the multiplexed OAM beam is transformed to *l*−*l* = 0, resulting in a bright spot at the beam center. In contrast, other OAM states $|{l}_{o}{{\rangle }}$ turns to *l*_0_−*l* ≠ 0, meaning they do not converge at the center. As shown in Fig. 5, the OAM modes at different positions are as follows: at diffraction order (−1,1), the OAM orders are 2, −1; at (1,1), the OAM orders are 3, −1, −5; at (−1, −1), the OAM orders are 5, 1, −3; at (1, −1), the OAM orders are 1, −2. This demonstrates the precise modulation of the OAM modes.
vi. *Amplitude*: Fig. 6 shows the amplitude analysis results, represented using the TAM spectrum. Schemes for TAM spectrum measurement have been widely reported in previous studies^40–44^. Here, we employ the mode projection for the analysis (see Supplementary Note 4 for more details). At diffraction order (−1,1), the MSE is 0.0032; at (1, 1), the MSE is 0.0032; at (−1, −1), the MSE is 0.0037; at (1, −1), the MSE is 0.0045, yielding a total average MSE of 0.0036 for the angular momentum spectrum measurement. Such successful experimental results highlight the remarkable performance of the proposed compact multiple DoFs high-dimensional manipulation.

## Discussion

This work presents a compact, high-dimensional, multiple DoFs tailoring scheme achieving an on-demand manipulation over wave vector, initial phase, spatial mode, OAM, and SAM through a single metasurface, demonstrating in a 288-dimensional optical field at once. The proposed hybrid strategy advances in a phase-only property, which facilitates easy integration into various diffractive optical devices to cope with various scenario requirements. Proof-of-principle experiments conducted on a metasurface sample show promising results. Such successful implementation confirms that our proposal enhances the manipulation capabilities of DoFs and expands the dimensionality of optical fields. Notably, as the theoretical eigenstates of OAM and their corresponding amplitudes are infinite, this work could be further strengthened by exploiting more eigenstates. Our findings not only provide a mean for multiple DoFs and high-dimensional beams compact manipulation, but also pave the way for further on-chip high-dimensional photonics, offering substantial potential for multifunctional photonic devices in both classical and quantum applications.

## Materials and methods

### Metasurface fabrication

The compact metasurface in our demonstration is fabricated by electron beam etching on a silica substrate with the diameter of 1 inch coated with a silicon film. A 1020-nm-thick silicon epitaxial layer was grown on a silica substrate using chemical vapor deposition (CVD). The silicon cuboids were patterned by electron beam lithography according to the designed slow-axis orientation angle distribution. The effective area of the structure is 700 μm × 700 μm, with an etch depth of 1020 nm, a minimum line spacing of 650 nm, and a minimum line width of 240 nm. The processing error is maintained below 5%.

### Experimental details

A 1617 nm distributed feedback (DBF) laser diode is employed as the source to generate Gaussian beams. To convert the beam into linear polarization, it first passes through a half wave plate (HWP) followed by a polarizer (P1). The spatial light modulator (SLM, Holoeye, PLUTO-TELCO-013-C) encodes a zero phase and acts as a reflector during the tailoring process. The incident linear polarization beam is focused onto the metasurface using a lens with a focal length of 300 mm. An infrared CCD camera (Cobra2000-CL1280-130vt-00, LUSTER) is then employed to capture the intensity distribution of the tailored beam. For evaluating the performance of circular polarization incident, an additional quarter-wave plate (QWP1) is placed between the lens and the metasurface, with fast axis arranged at −45° or 45°. For initial phase analysis, an additional polarizer (P2) is placed between metasurface and CCD camera, with main axis oriented sequentially at 0°, 45° and 90°. For TAM spectrum measurement, a quarter-wave plate (QWP2) and a polarizer (P2) are introduced between metasurface and CCD camera to separate the SAM components. The fast axis of QWP2 is arranged at 45° while the main axis of P2 is arranged at 0° and 90° sequentially. For each SAM components, the SLM encodes a series of anti-spiral phases (−*l*_1_, −*l*_2_, …, −*l*_N_), and the intensity of the central bright spot is calculated to represent the amplitude of the OAM mode *l*_1_, *l*_2_, …, *l*_N_.

## Supplementary information

Supplementary Information for Hybrid strategy in compact tailoring of multiple degrees-of-freedom toward high-dimensional photonics

## Data Availability

The data that support the findings of this study are available within the paper and the supplementary. Additional data related to this paper are available from the corresponding authors upon reasonable request.

## Code availability

The code that supports the findings of this study are available from the corresponding author upon reasonable request.
