# Supplementary material for: Hybrid strategy in compact tailoring of multiple degrees-of-freedom toward high-dimensional photonics
Source: Light Sci Appl. 2025 Apr 21;14:167. doi: 10.1038/s41377-025-01857-3 (PMC12012099; doi:10.1038/s41377-025-01857-3)
Supplement: Supplementary file 1 — Supplementary Information for Hybrid strategy in compact tailoring of multiple degrees-of-freedom toward high-dimensional photonics [file 41377_2025_1857_MOESM1_ESM.docx]

**Supplementary Information for**

**Hybrid strategy in compact tailoring of multiple degrees-of-freedom toward high-dimensional photonics**

Shiyun Zhou^1,2,3^, Lang Li^1,2,3^, Liliang Gao^1,2,3^, Zhiyuan Zhou^4,5^, Jinyu Yang^1,2,3^, Shurui Zhang^1,2,3^, Tonglu Wang^6^

Chunqing Gao^1,2,3,7^ and Shiyao Fu^1,2,3,7 *^

*^1^ School of Optics and Photonics, Beijing Institute of Technology, Beijing 100081, China*

*^2^ Key Laboratory of Photoelectronic Imaging Technology and System, Ministry of Education of the People’s Republic of China, Beijing 100081, China*

*^3^ Key Laboratory of Information Photonics Technology, Ministry of Industry and Information Technology of the People’s Republic of China, Beijing 100081, China*

*^4^ Key Laboratory of Quantum Information, University of Science and Technology of China, Hefei 230026, China*

*^5^ Synergetic Innovation Center of Quantum Information & Quantum Physics, University of Science and Technology of China, Hefei 230026, China*

*^6^ Beijing National Research Center for Information Science and Technology, School of Integrated Circuits, Tsinghua University, Beijing 100084, China*

*^7^ National Key Laboratory on Near-surface Detection, Beijing, 100072, China*

*Correspondence: fushiyao@bit.edu.cn*

**Supplementary Note 1: AI-assisted model training**

The training dataset for the neural network generating superimposed OAM mode phase consists of simulated data, matching experimental parameters at a wavelength of 1617nm and a waist size of 0.5mm. Each data pair includes a target OAM spectrum along with the corresponding intensity and phase patterns, totaling 3052 pairs. The dataset is divided into training and testing sets in an 8:2 ratio to ensure robust validation. During training, we employ the Adam optimizer with a learning rate of 0.001 and a batch size of 8, spanning 800 epochs. Initially, the network parameters are optimized to converge efficiently, and after pre-training, fine-tuning is applied by specifically adapting the last few layers to our target task. Additionally, learning rate schedules are implemented to further mitigate overfitting, and an early stopping callback is used to terminate training if the validation loss remains unchanged for 5 consecutive epochs. The network training and testing are conducted using the PyTorch framework in Python3.8 on a workstation equipped with four NVIDIA RTX A6000 Graphical Processing Unit (GPU), an Intel(R) Xeon(R) E5-2699 v4 CPU running at 2.20GHz, and 192 GB of RAM, all on Ubuntu 20.04.6 LTS. The entire training process took approximately 40 hours. Testing example results, corresponding to superimposed OAM modes ranging from a maximum of 11 modes to a minimum of 3 (covering the OAM range from -5 to 5), are presented in Figure S1.


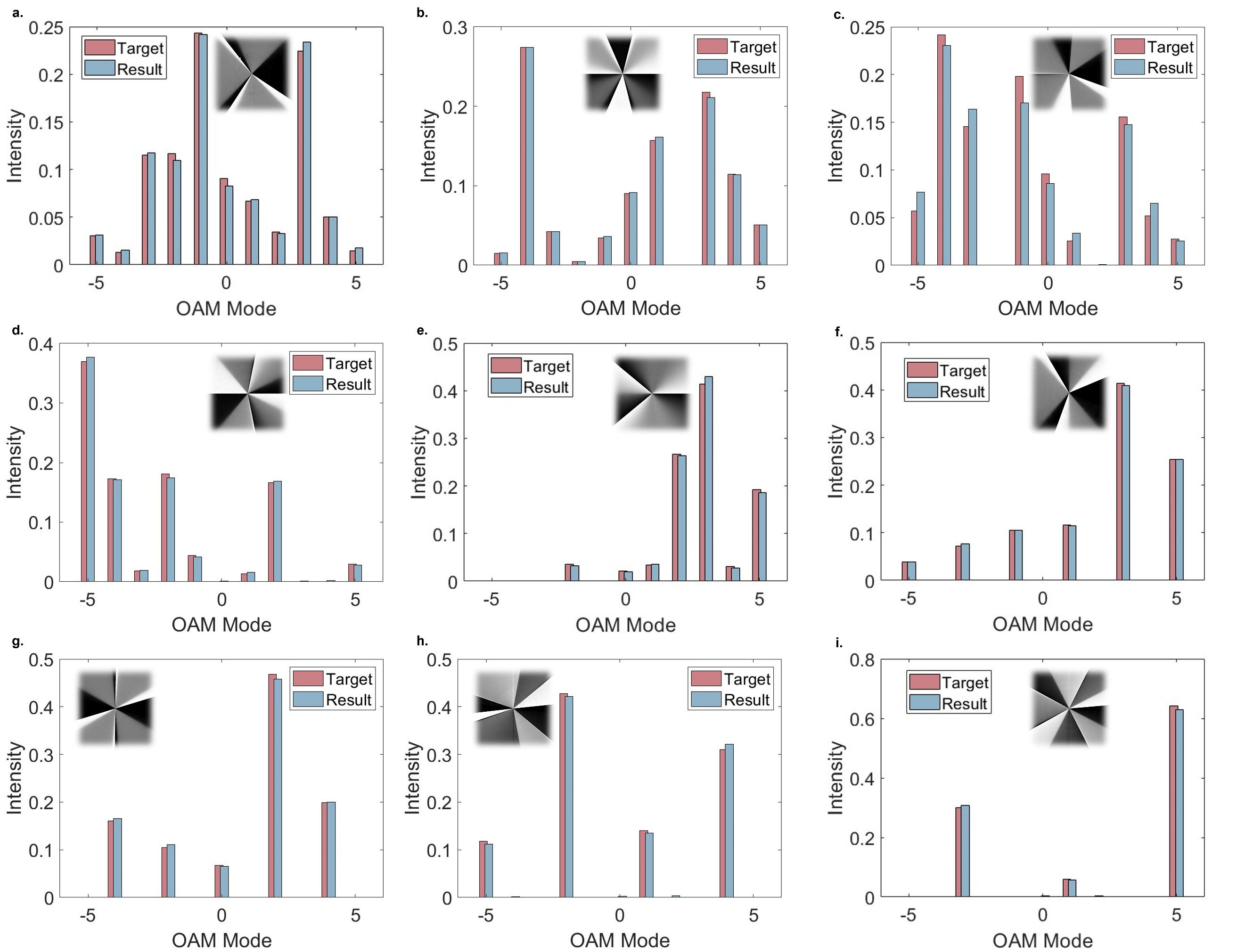


**Figure S1.** Testing example results. From (a) to (i) display the superimposed OAM modes phase result, ranging from a maximum of 11 modes to a minimum of 3, covering the OAM mode from -5 to 5.

**Supplementary Note 2: Metasurface optimization**

We simulate the silicon cuboid used in our metasurface with the Lumerical FDTD Solutions software. The cuboid structure is positioned on a substrate along the +z direction, with a plane wave source directed upwards from below the SiO₂ substrate. Transmitted fields are captured by a field monitor placed above the structures. For the silicon cell simulation, we applied periodic boundary conditions along the x and y axes, and perfectly matched layers along the z axis. By rotating the silicon cuboid, we analyzed phase shift and transmission efficiency at the operational wavelength, as shown in Figures S2a and S2b, with the height ($h_{\mu}$) varying from 850nm to 1050nm. The ideal unit is capable of achieving phase modulation from -π to π with high transmittance during rotation. Considering fabrication challenges, we selected $h_{\mu}=1020nm$, where the simulated phase modulation and transmittance performances of the metasurface meet our requirements. Each unit cell is designed to function as a miniature half-wave plate, with the optimal phase difference ($\Delta\Phi$) between the y and x polarizations set to π. We then adjusted the length ($l_{\mu}$) and width ($w_{\mu}$) to accomplish the requirement, with $\Delta\Phi$ calculated for $l_{\mu}$ scanning from 450nm to 550nm and$w_{\mu}$ from 200nm to 300nm, as shown in Figure S2c. The optimal dimensions for the silicon cuboid are $l_{\mu}=513.3nm$, $w_{\mu}=214.3nm$.


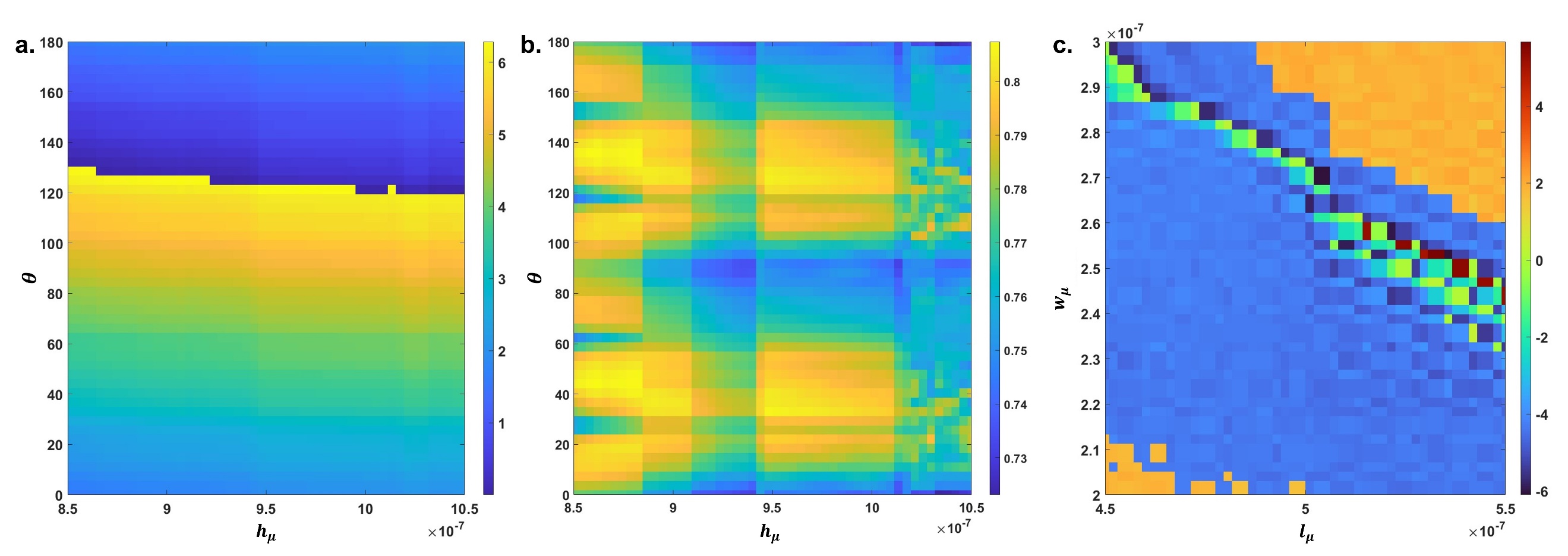


**Figure S2.** Evaluation of the demonstration compact metasurface. **a)** and **b)** are phase modulation and transmission efficiency, respectively, versus heights and rotation degrees (*θ*) of a silicon cuboid unit. **c)** is phase difference (Δ*Φ*) between the *y* and *x* polarizations of the designed metasurface versus widths and lengths of a silicon cuboid unit.

**Supplementary Note 3: Initial phase calculation**

The determination method of the rotation angles is inspired by the log-polar transformation, the specific steps of it are as follows:

i) Move the two patterns to be compared at the center of the images, subtracting background and undergoing normalization.

ii) Draw rays from the center of the image to the edges at various angles. (The angle of the horizontal ray pointing to the right is denoted as 0 degrees, and the angular difference between adjacent ray is 360/N, where N is the resolution.)

iii) For each angle, integrate the pixel value covered by each ray on the image. Plot the polar coordinate gray curve. (The angle corresponding to each ray as the horizontal axis and the pixel integral value as the vertical axis.)

iv) Circular shift the second curve along the horizontal axis with t-step, and calculate the Hadamard product Ir(t) between it and the first curve. When Ir(t) reaches its maximum, it indicates that the similarity between the two curves is the highest. Denote it as Ir(tm).

v) The rotation angle of these two patterns is calculated as 360×tm/N.


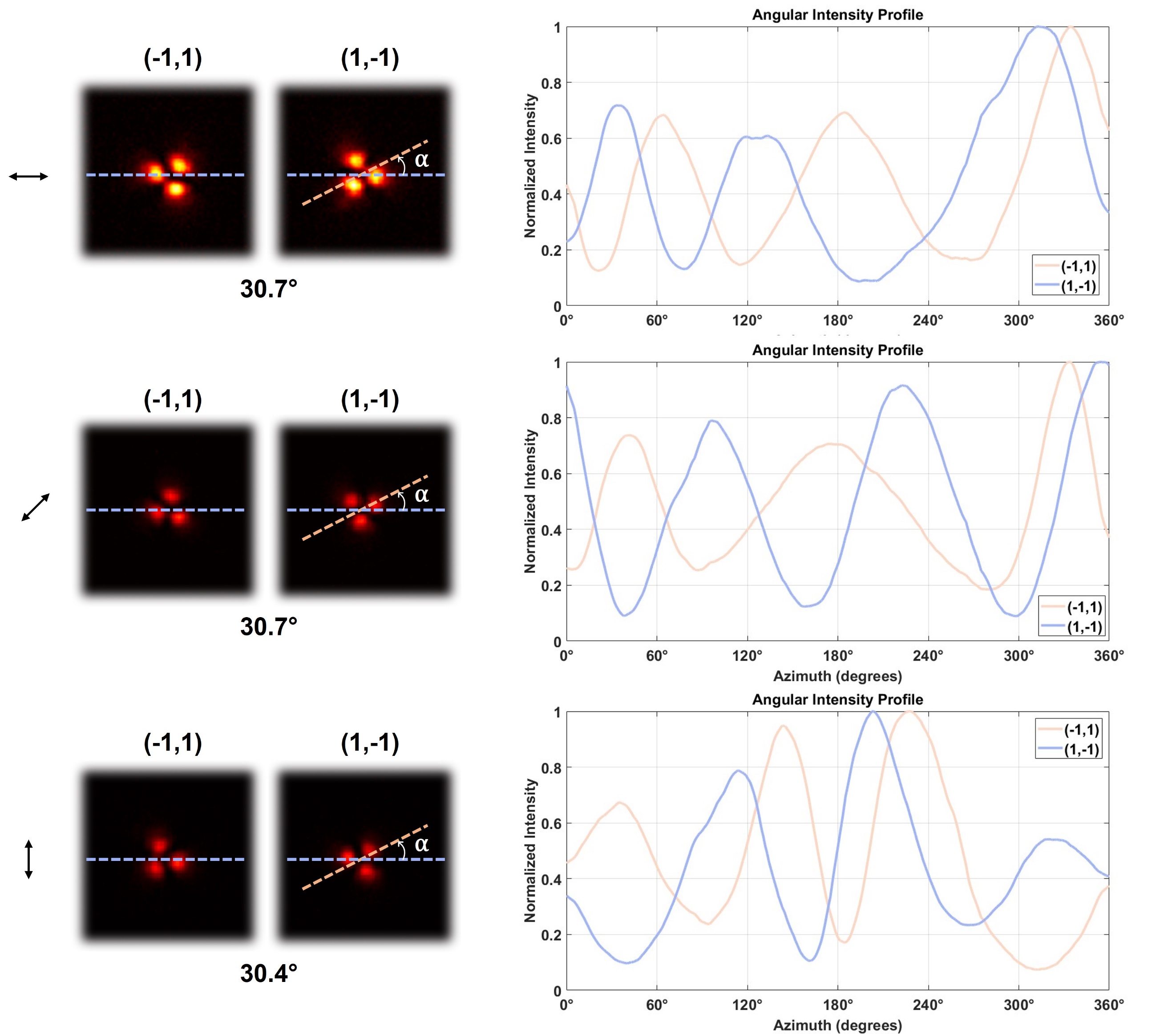


**Figure S3.** Experimentally calculation results of rotational angle for initial phase verify. Measurements under 0°, 45°, and 90° polarizations yielded rotation angle of 30.7°, 30.7°, and 30.4°, respectively. Resulting in an average rotation angle of 30.6°.

**Supplementary Note 4: Total angular spectrum measurement**

The quantitative analysis first using the QWP to separate SAM DoF, then, the spectrum measurement of multiplexed OAM beam to determine the amplitude distribution is performed through OAM back conversion, which involves applying a series of anti-spiral phases to the beams. The resulting back-converted patterns are then captured by a CCD camera. The principle of back conversion can be explained as follows: if a $-l$-th order spiral phase is encoded, the OAM state $l$ in the multiplexed OAM beam is transformed to $l-l=0$, resulting in a bright spot at the beam center. In contrast, other OAM states $l_{o}$ turns to $l_{o}-l\neq0$, meaning they do not converge at the center. Therefore, by measuring the intensity of the bright spot in each back-converted pattern, we can determine the OAM modes and their amplitude. Figure S3 shows the experimentally captured back-converted patterns of LCP at (1,1) and RCP at (-1, -1), with the orders of the back-converting spiral phases labeled. The green dashed circle in each inset is the sampling area, where the intensities are considered as the back-converted OAM channel. It is worth noting that various methods can be utilized for this quantitative analysis, including log-polar coordinate transformer [43] and the rotational Doppler effect [44], both of which are viable alternatives.


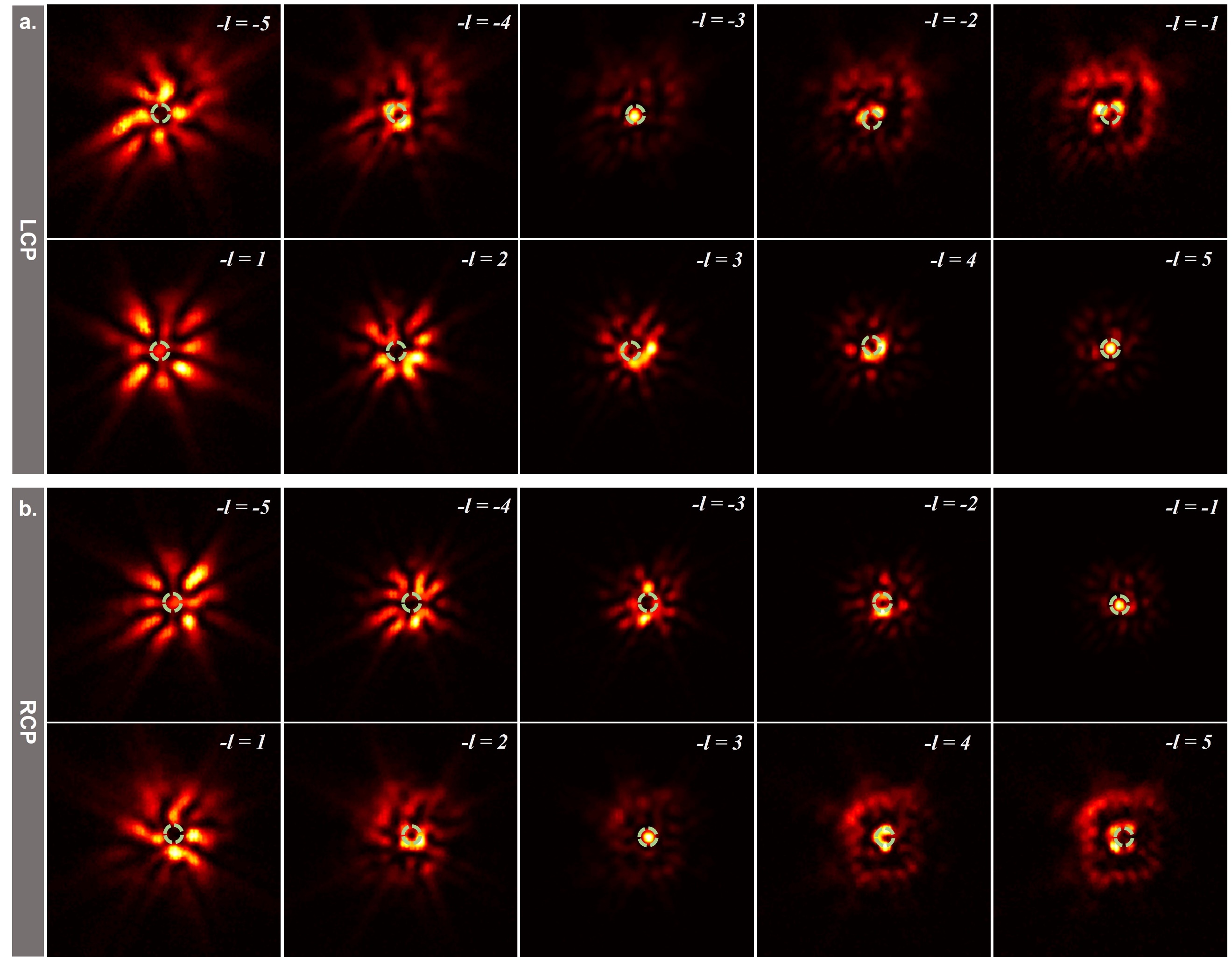


**Figure S4.** Experimentally captured back-converted patterns of LCP at (1,1) and RCP at (-1, -1). The orders of the back-converting spiral phases are labeled at the top right corner of each inset. The green dashed circle represents the sampling area, where intensities inside it are regarded as the back-converted OAM channel.

**Supplementary Note 5: Discussion in scheme scalability**

To discuss the scalability of our proposed hybrid strategy scheme, we begin by testing the effect of increasing OAM and its amplitude, which serve as high-dimensional DoFs. Simulation results displayed in Fig. S5 indicate that our proposal can efficiently handle OAM modes within the range of -5 to 5, from maximum superposed OAM mode 11 to the basic demonstration 3 (from Fig. S5(a) to (i)). In fact, our AI-based tailoring scheme for multiplexed OAM mode can support an OAM range from -75 to 75. However, as the OAM order increases, the intensity pattern expands. Therefore, the OAM tailoring dimension is constrained by the limitations of the receiving plane.


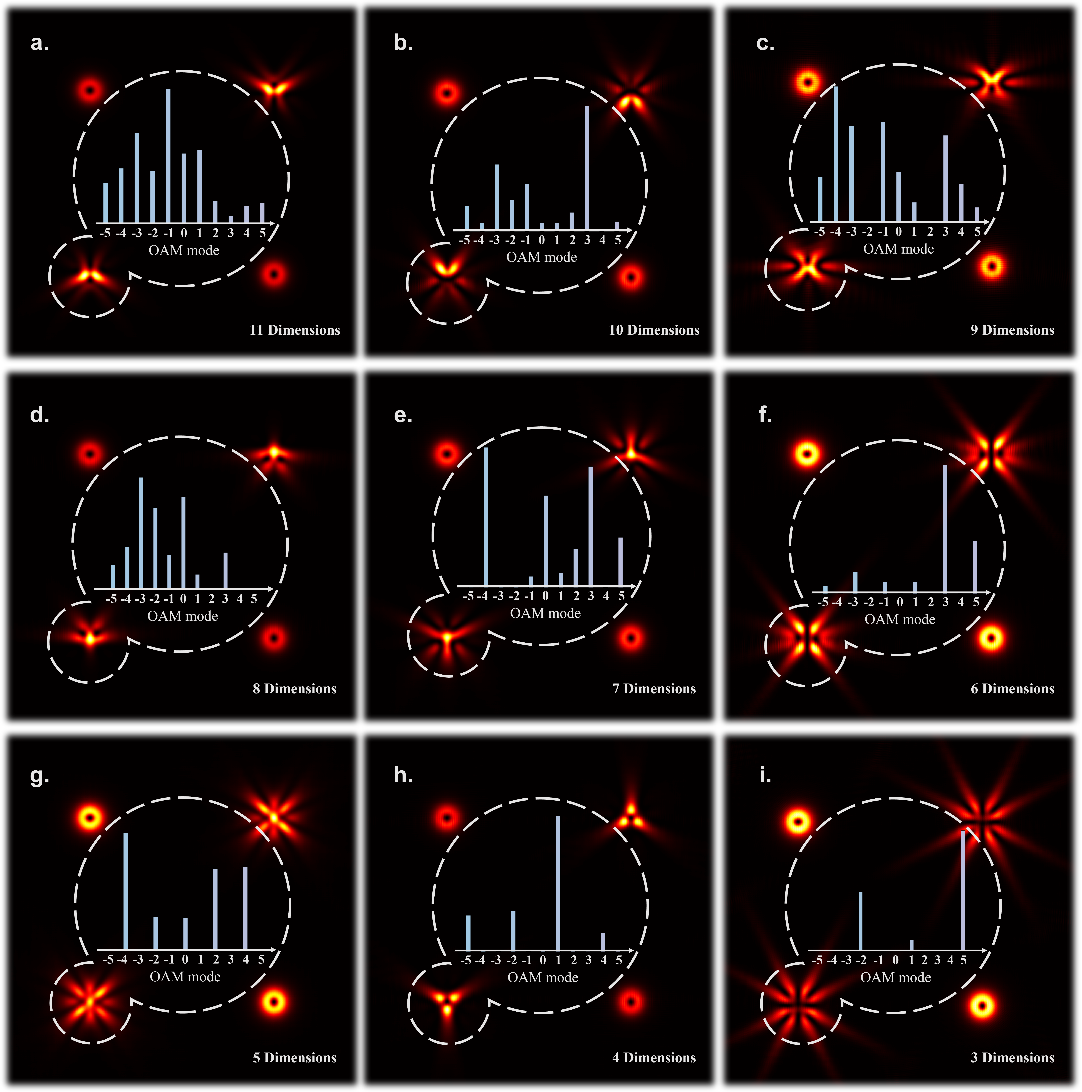


**Figure S5.** The scalability of OAM and amplitude dimensions. (a)-(i) simulation results for OAM modes ranging from -5 to 5, covering from the maximum superposed OAM mode 11 to the most basic 3. The corresponding amplitude distributions are also displayed.

Further scalability analysis (Fig. S6) shows that increasing the wave vector dimension from 4 to higher configurations (e.g., 8, 12, 16) is feasible. However, as wave vector dimension increases, cross-talk between diffraction orders becomes more pronounced, ultimately limiting the theoretical capacity of the wave vector dimension. These findings highlight the scalability of our intelligent hybrid strategy scheme in a truly high-dimensional tailoring, with its primary limitations stemming from the physical constraints of the receiving plane and diffraction-related cross-talk.


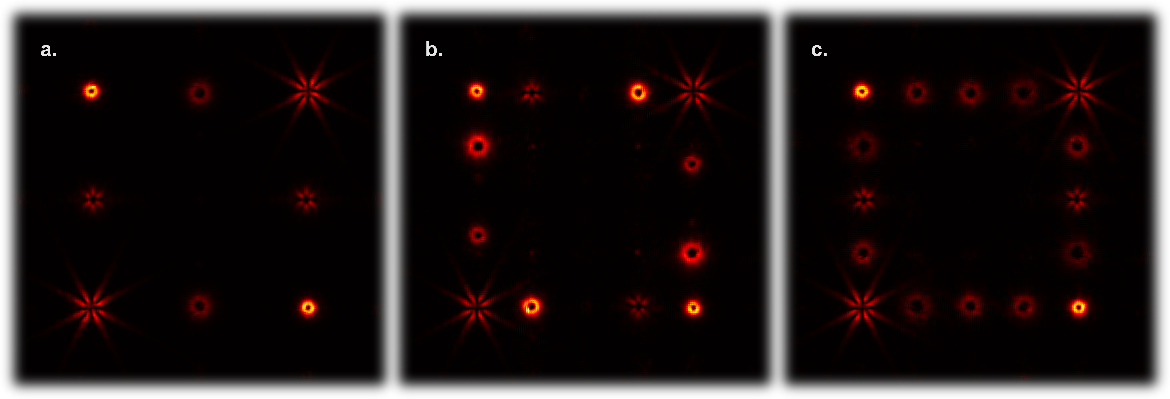


**Figure S6.** The scalability of wave vector dimensions. (a) 8-dimensional tailoring of the wave vector; (b) 12-dimensional tailoring of the wave vector; (c) 16-dimensional tailoring of the wave vector;

**Supplementary Note 6: Ablation study on the phase grid resolution**

In order to investigate the impact of phase grid resolution on the performance of our proposed intelligent hybrid strategy scheme, we conduct a series of tests using various grid resolutions. The phase grid resolution used in this study is 1080×1080, which matches the experimental setup utilizing a liquid crystal spatial light modulator. The effect of different resolutions was tested in a range from 1080×1080 to 237×237, with the following configurations: (a) 1080×1080, (b) 720×720, (c) 540×540, (d) 238×238, and (e) 237×237, as shown in Fig. S7. The results show that our method achieves a high level of modulation even at the lowest tested resolution of 238×238, indicating the robustness of the approach. Notably, although lower resolutions lead to more compact patterns, the modulation performance remains relatively unaffected. This ablation study indicates that our method can efficiently handle low-resolution grids while still delivering reliable results. The relationship between phase grid resolution and modulation quality can be understood by considering the ideal phase distribution. Typically, the ideal phase distribution is continuous, and the modulated phase generated by our method represents a discrete sample of this continuous phase. Our approach demonstrates considerable flexibility, retaining its effectiveness across a wide range of resolutions. These findings highlight the significant robustness of our scheme with respect to phase grid resolution, offering valuable flexibility for practical implementation. The ability to perform modulation at lower resolutions makes the approach highly adaptable and suitable for various real-world applications, where hardware limitations or computational constraints might necessitate lower grid resolutions.


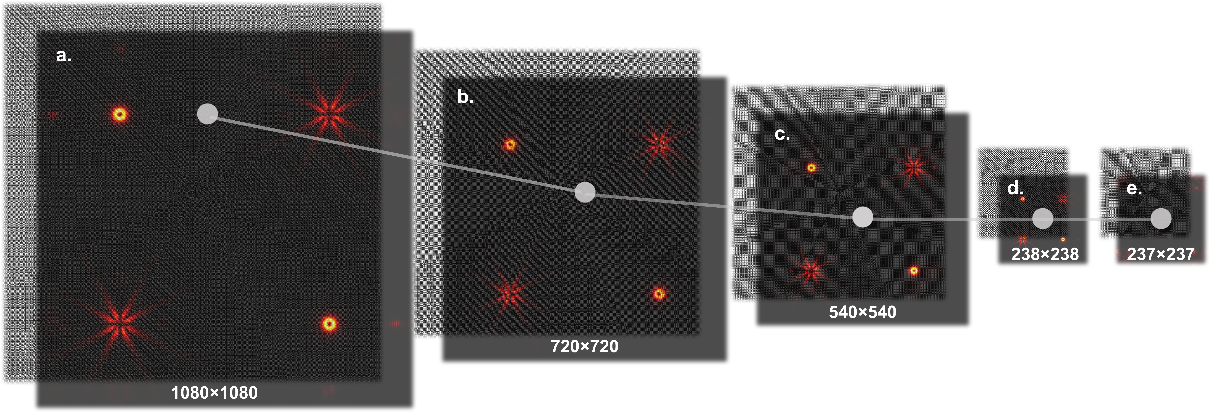


**Figure S7.** Test with varying phase grid resolution. (a) Results under grid resolution 1080×1080 (used in manuscript); (b) Results under grid resolution 720×720; (c) Results under grid resolution 540×540; (d) Results under minimum effective grid resolution 238×238; (e) Results under grid resolution 237×237.
